# Effects of Phytoestrogen Extracts Isolated from Elder Flower on Hormone Production and Receptor Expression of Trophoblast Tumor Cells JEG-3 and BeWo, as well as MCF7 Breast Cancer Cells

**DOI:** 10.3390/nu8100616

**Published:** 2016-10-08

**Authors:** Lennard Schröder, Dagmar Ulrike Richter, Birgit Piechulla, Mareike Chrobak, Christina Kuhn, Sandra Schulze, Sybille Abarzua, Udo Jeschke, Tobias Weissenbacher

**Affiliations:** 1 Department of Obstetrics and Gynaecology, Ludwig-Maximilians-University of Munich, Munich 80337, Germany; lennard.schroeder@med.uni-muenchen.de (L.S.); Christina.kuhn@med.uni-muenchen.de (C.K.); sandra.schulze@med.uni-muenchen.de (S.S.); tobias.weissenbacher@med.uni-muenchen.de (T.W.); 2 Department of Obstetrics and Gynaecology, University of Rostock, Rostock 18059, Germany; dagmar.richter@kliniksued-rostock.de; 3 Department of Biological Sciences, University of Rostock, Rostock 18059, Germany; birgit.piechulla@uni-rostock.de (B.P.); chrobak@bni-hamburg.de (M.C.); sybille.abarzua@uni-rostock.de (S.A.)

**Keywords:** lignans, isoflavones, elder flower, breast cancer, trophoblast tumor

## Abstract

Herein we investigated the effect of elderflower extracts (EFE) and of enterolactone/enterodiol on hormone production and proliferation of trophoblast tumor cell lines JEG-3 and BeWo, as well as MCF7 breast cancer cells. The EFE was analyzed by mass spectrometry. Cells were incubated with various concentrations of EFE. Untreated cells served as controls. Supernatants were tested for estradiol production with an ELISA method. Furthermore, the effect of the EFE on ERα/ERβ/PR expression was assessed by immunocytochemistry. EFE contains a substantial amount of lignans. Estradiol production was inhibited in all cells in a concentration-dependent manner. EFE upregulated ERα in JEG-3 cell lines. In MCF7 cells, a significant ERα downregulation and PR upregulation were observed. The control substances enterolactone and enterodiol in contrast inhibited the expression of both ER and of PR in MCF7 cells. In addition, the production of estradiol was upregulated in BeWo and MCF7 cells in a concentration dependent manner. The downregulating effect of EFE on ERα expression and the upregulation of the PR expression in MFC-7 cells are promising results. Therefore, additional unknown substances might be responsible for ERα downregulation and PR upregulation. These findings suggest potential use of EFE in breast cancer prevention and/or treatment and warrant further investigation.

## 1. Introduction

A growing body of data points to health benefits of phytoestrogens in diet and to possible pharmaceutical applications [1]. The two main groups of phytoestrogens, isoflavones and lignans, are polyphenolic compounds derived from plants with a molecular structure that closely resembles mammalian estrogens [2]. Due to their molecular structure, these compounds can bind and interact with human estrogen receptors (ER) resulting in both estrogen and anti-estrogen effects [3]. Thus, it is assumed that some phytoestrogens can be classified as selective estrogen receptor modulators (SERM) [4,5]. 

Isoflavones are mostly found in legumes, with the most common representative being soy and its derivative products [6], making them more common in Asian diets, whereas lignans, more common in occidental diets, are usually found in seeds and fiber-rich cereals [7,8]. Their role in the pathogenesis of hormone-dependent malignancies, especially breast cancer, has been investigated using chemically pure isolates or product extracts in several in vitro or in vivo models [9,10,11]. Their effects as hormonally-active diet components have been excessively and controversially discussed [12,13]. Isoflavone extracts and supplements are often used for the treatment of menopausal symptoms and for the prevention of age-associated conditions, such as cardiovascular diseases and osteoporosis in postmenopausal women [12]. 

In humans the most important lignans are secoisolariciresinol and matairesinol [14]. 

After oral intake they are transformed by intestinal aerobe and anerobe flora into bioavailable enterolignans enterodiol and enterolactone [15].

Clinical studies proved that a high exposition to enterolignans reduced the risk of breast cancer by 16% [16]. Moreover, increased blood concentrations of enterolactone in postmenopausal women are related with a significant reduction of breast cancer mortality [17].

With the goal of identifying potential sources of phytoestrogens and selecting those with beneficial functions, our group has tested, in prior trials, the phytoestrogen properties of pumpkin and flax seed lignan and isoflavone extracts on the proliferation of trophoblast and breast cancer cell lines [18,19]. Moreover, the effect of the phytoestrogens genistein and daidzein on human term trophoblasts and their influence on fertility was investigated [20].

Elder flower (*Sambucus nigra*) is a historically-significant herbal medicinal plant used for centuries as a cold remedy. It is used as a general nutritive tonic and due to its strong taste as a flavor enhancer in meals and beverages. Elder extracts possess significant antioxidant activity and have been shown to impair angiogenesis. The anthocyanins present in elderberries protect vascular epithelial cells against oxidative insult, and reduce low-density lipoprotein (LDL) and cholesterol, therefore, preventing vascular disease [21]. Elder extracts boost cytokine production [22]. The influenza A virus subtype H1N1 inhibition activities of the elder flavonoids compare favorably to the known anti-influenza activities of oseltamivir and amantadine [23]. The terpenes extracted from elder flower show notably strong antimicrobial effects in vitro upon methicillin-resistant *Staphylococcus* *aureus* [24]. Moreover elder flower could improve bone properties by inhibiting the process of bone resorption and stimulating the process of bone formation [25].

Due to the interesting characteristics of elder flower described above, this in vitro study aims to identify the distribution of lignans and isoflavones in elder flower extracts (EFE) and evaluate the potential phytoestrogen effects of EFE on tumor trophoblast BeWo and JEG-3 cells and the ER-positive MCF7 breast cancer cell lines, and compare those with the effects of enterodiol and enterolactone. 

## 2. Materials and Methods

### 2.1. Preparation of the EFE

In total six EFE from the species *Sambucus nigra* were produced. Three lignan-isolations were prepared as previously described [26] and, afterwards, dissolved in 100% ethanol. In the aim to verify the previously-reported increased lignan concentration in elder flowers [27] the molecular–chemical composition of the extract was further analyzed by pyrolysis-field ionization mass spectrometry by using an LCQ-Advantage (Thermo Finnigan’s, Arcade, NY, USA). The peaks were identified by ion trap technology in electrospray ionisation (ESI) mode. The source voltage was set at 4.5 kV, while the mass detection range was 150–2000 amu. For the production of the three flavonoid extracts, the method previously described by Franz and Koehler was used [28].

### 2.2. Cell Lines

For the current work the chorion carcinoma cell lines JEG-3 and BeWo, and the breast carcinoma cell line MCF7, were used. All cell lines were obtained from the European Collection of Cell Cultures (ECACC, Salisbury, UK). The cells were grown in Dulbecco’s Modified Eagle Medium (DMEM) without phenol red (Biochrom AG, Berlin, Germany) supplemented with 10% heat-inactivated fetal calf serum (PAA Laboratories GmbH, Pasching, Austria), 100 μg/mL Penicillin/Streptomycin (Biochrom AG) and 2.5 μg/mL Amphotericin B (Biochrom AG). Cultures were maintained in a humidified incubator at 37 °C with a 5% CO_2_ atmosphere. Prior to cell culture, the levels of estrogen or progesterone in the medium were measured, using an automated Immulite (DPC Biermann, Freiburg, Germany) hormone analyzer, in order to exclude their presence. 

### 2.3. Effect of EFE on Cell Lines

For all experiments, the cells were seeded on Quadriperm tissue slides with or without added lignan and flavonoid EFE separately. In brief, cells were seeded at a concentration of 400,000 cells per slide. The cells were left to attach for 24 h. Then, the medium was replaced by medium supplemented with lignan and flavonoid EFE separately at final effective concentrations of 10, 50, and 100 μg/mL. Since the original EFE was diluted in 100% ethanol, medium supplemented with 100% ethanol at a concentration of 5 μg/mL (this being the maximum ethanol concentration achieved during these experiments) served as the internal control. In addition, enterolactone and enterodiol (Sigma-Aldrich, Taufkirchen, Germany) were added to the same cell cultures as used for EFE in concentrations of 10, 50, and 100 μg/mL, respectively. After the cells were cultured for 72 h, 1 mL from each supernatant was stored at −80 °C for estradiol analysis. The remaining supernatant was then discarded and the slides were washed in phosphate-buffered saline (PBS), fixed in acetone for 10 min, and left to dry at room temperature. Cells treated with equal concentrations of estradiol (10, 50, and 100 μg/mL) served as external controls.

### 2.4. Estradiol Determination in the Cell Culture Medium

For the determination of estradiol in the culture medium, a competitive enzyme immuno-assay (EIA) was applied as described previously [29]. The measurements were performed using an automated Immulite 2000 (DPC Biermann, Freiburg, Germany) hormone analyzer.

### 2.5. Immunocytochemistry for the ER*α*, ER*β*, and Progesterone Receptor (PR)

For immuno-detection of the steroid receptors ERα, ERβ, and PR, the Vectastain R Elite Avidin/Biotin Complex (ABC) Kit (Vector Laboratories, Burlingame, CA, USA) was used according to the manufacturer’s protocol. After being air dried, the slides were rinsed in PBS for 5 min and incubated with the ABC normal serum for 60 min in a humidified environment. The slides were then washed and incubated with the respective primary antibodies. Salient features of the antibodies used are presented in Table 1. The slides were then incubated with the diluted biotinylated secondary antibody (30 min), followed by incubation with the ABC reagent (30 min), and the ABC substrate (15 min). A PBS wash (5 min) was applied between steps. Finally, the slides were counterstained with Mayer’s acidic hematoxylin (30 s), rinsed with water, and covered with Aquatex. The intensity and distribution patterns of the specific immunocytochemical staining was evaluated using a semi-quantitative method (IRS score) as previously described [30]. Briefly, the IRS score was calculated as the product of the optical staining intensity (0 = no staining; 1 = weak staining; 2 = moderate staining; and 3 = strong straining) multiplied by staining extent (0 = no staining; 1% ≤ 10% staining; 2 = 11%–50% staining; 3 = 51%–80% staining and 4 ≥ 80% staining). The percentage of positively-stained cells was estimated by counting approximately 100 cells.

### 2.6. Statistical Analysis

The results are presented as mean ± sem of three independent experiments. Statistical analysis was performed using the Wilcoxon’s signed rank tests for pairwise comparisons. Each observation with *p* < 0.05 was considered statistically significant.

## 3. Results

### 3.1. EFE Contains Phytoestrogen Compounds

**Figure 1:**
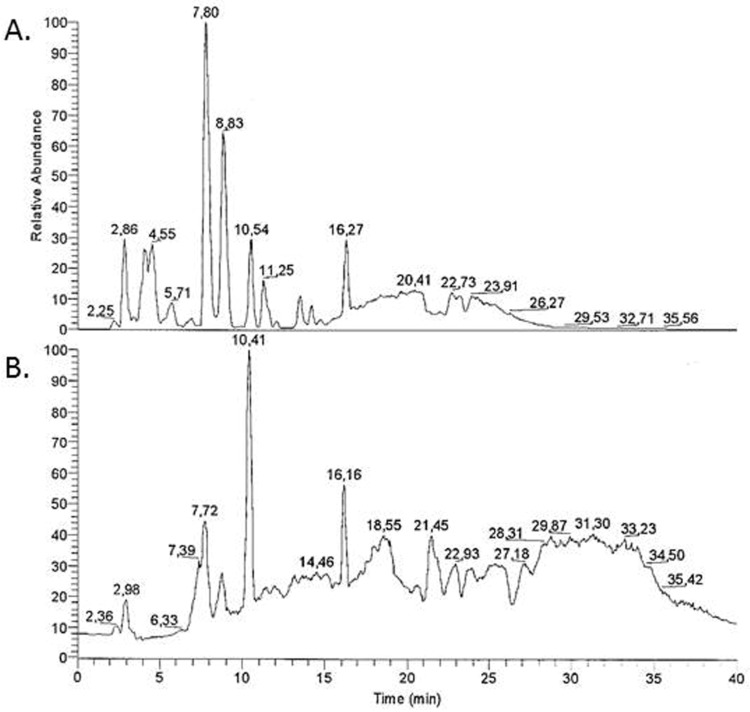

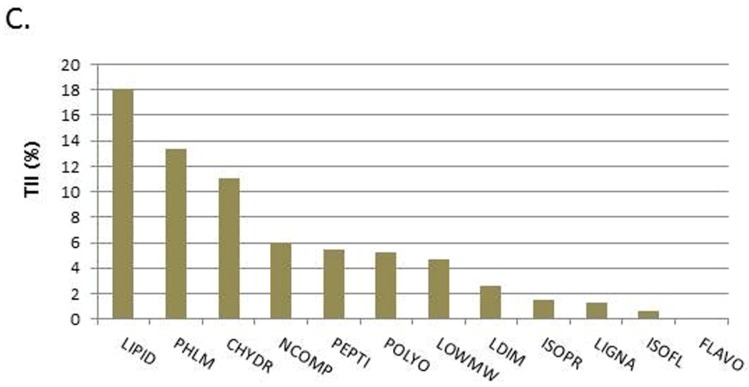
Characteristic diagram of mass spectrometry analysis results of the EFE using both the microwave extraction (**A**) and the extraction method modified from Luyengi et al. [26] (**B**); moreover, the different substances extracted are presented (**C**).

Mass spectrometry was performed to identify the different substrates and to determine their proportions in EFE. The results showed that the EFE contains phytoestrogen compounds. Lignan dimers (LDIM) were found with a total intensity of 2.6%, lignans (LIGNA) with 1.3%, isoflavones (ISOFL) with 0.6%, and flavones (FLAVO) with 0.1%. Figure 1 demonstrates the distribution of the different substance classes found in EFE. With a total intensity of 18.1% the most abundant substance class in EFE were lipids, including alcanes, alcenes, fatty acids, waxes, and fats (LIPID). Monolignoles (PHLM) were found with an intensity of 13.4% and carbohydrates (CHYDR) with 11.1%. Nitrogen (NCOMP) compounds were found with a total intensity of 6%, amino acids and peptides with 5.4% (PEPTI), isoprenoid compounds (ISOPR) with 1.5%, other polyphenolic (POLYO) with 5.2%, and low molecular compounds (LOWMW) with 4.7%.

### 3.2. EFE Lignan and Flavonoid Extracts Induce the Inhibition of Estradiol Secretion in JEG-3, BeWo, and MCF7 Cells in a Dose-Response Pattern and the Inhibition of Progesterone Secretion in JEG-3 Cells

**Figure 2:**
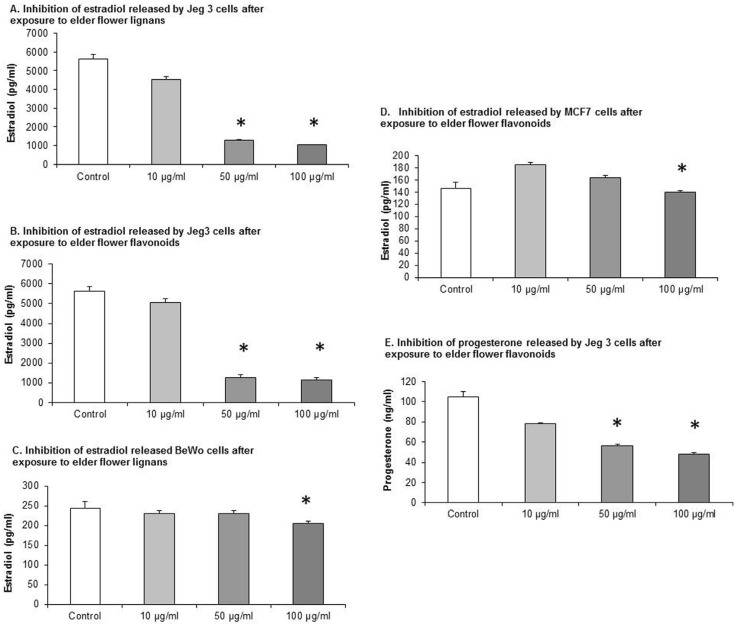
Estradiol and progesterone concentration in the tissue culture medium of JEG-3, BeWo, and MCF7 cells in the absence or presence of EFE. The effective EFE concentrations were 10 μg/mL, 50 μg/mL, and 100 μg/mL. Significantly different observations are highlighted with an asterisk.

To assess the estradiol and progesterone secretion, all three cell lines were cultured for 72 h in the presence of different EFE concentrations. An automated hormone analyzer was used to determine the estradiol and progesterone concentration in the medium by applying a competitive EIA. All cell lines were incubated with elder flower flavonoid and lignan extracts. The EFE lignan and flavonoid extracts demonstrated a statistical significant inhibition in estradiol secretion in a dose-response pattern in all three cell lines (Figure 2). Only statistical significant data is demonstrated in the figures. The cell culture medium with 10% FCS did not contain any measurable amounts of estrogen and progesterone, as determined with the automated hormone analyzer Immulite (DPC Biermann, Freiburg, Germany).

In JEG-3 cells, the estradiol production was inhibited from 5634.96 ± 235.77 pg/mL in the control to 4547.48 ± 145.89 pg/mL, 1283.88 ± 29.78 pg/mL, and 1030.43 ± 24.50 pg/mL when the EFE lignan concentration was 10 μg/mL, 50 μg/mL, and 100 μg/mL, *p* = 0.018, respectively (Figure 2A). EFE flavonoids had a similar effect using the same concentrations, as the estradiol production was inhibited from 5634.97 ± 235.77 pg/mL in the control to 5049 ± 187.28 pg/mL, 1264.5 ± 151.26 pg/mL, and 1137 ± 138.08 pg/mL (Figure 2B).

In JEG-3 cell lines progesterone secretion was also significantly inhibited using EFE lignan extracts from 87.95 ± 1.36 pg/mL in the control to 84.88 ± 1.98 pg/mL, 66.22 ± 2.25 pg/mL, and 45.98 ± 1.92 pg/mL when the EFE concentration was 10 μg/mL, 50 μg/mL and 100 μg/mL. 

The cultivation of the BeWo cell line with EFE lignan extracts resulted again in an inhibition of estradiol secretion from 245.25 ± 16.25 pg/mL in the control to 230.85 ± 8.17 pg/mL, 231.95 ± 6.1 pg/mL, and 206.81 ± 5.69 pg/mL when the EFE concentration was 10 μg/mL, 50 μg/mL, and 100 μg/mL (Figure 2C). The differences between the stimulated cells and the control were only significant at a concentration of 100 μg/mL, with *p* = 0.05. 

In MCF7 cell lines the EFE flavonoid concentrations of 10 μg/mL and 50 μg/mL first provoked a transient increased secretion of estradiol from 146.37 ± 9.91 pg/mL in the control to 185.44 ± 4.28 pg/mL at 10 μg/mL and 164.07 ± 3.16 pg/mL at 50 μg/mL (Figure 2D). Then, at 100 μg/mL, the estradiol secretion was inhibited to 140.21 ± 2.22 pg/mL, *p* = 0.08 respectively. 

Using the same concentrations with EFE flavonoid-extracts, progesterone secretion was also significantly inhibited in JEG-3 cells (Figure 2E) from 104.83 ± 5.13 pg/mL in the control to 77.94 ± 1.32 pg/mL, 56.18 ± 1.7 pg/mL, and 47.76 ± 1.56 pg/mL (*p* = 0.043).

### 3.3. EFE Flavonoid Extracts up Regulates ER*α* in JEG-3 Cells

**Figure 3:**
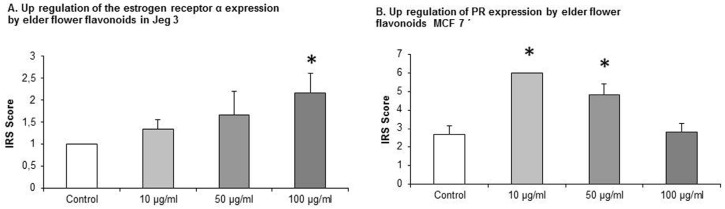
Upregulation of ER α and progesterone receptor by elder flower flavonoids in JEG-3 and MCF7 cells. The effective EFE concentrations were 10 μg/mL, 50 μg/mL, and 100 μg/mL. Significantly different observations are highlighted with an asterisk.

JEG-3 cell lines that were cultivated with EFE flavonoid in the concentrations of 10 μg/mL, 50 μg/mL, and 100 μg/mL an upregulation of ERα was demonstrated. The IRS score of ERα was increased from 1 ± 0 in the control to 1.33 ± 0.23, 1.67 ± 0.54, and 2.167 ± 0.44. At 100 μg/mL statistical significance was demonstrated, *p* = 0.015, respectively (Figure 3A). 

### 3.4. EFE Flavonoids Downregulate ER *α* and EFE Lignans and Flavonoids Upregulate the PR in a Dose-Response Pattern Predominantly in Lower Concentrations in MCF7 Cells

**Figure 4:**
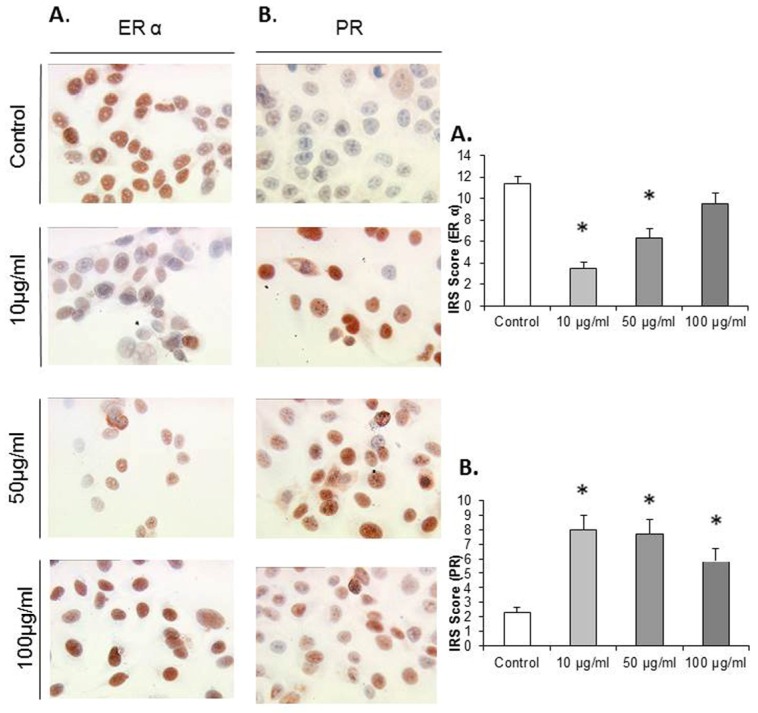
Representative microphotographs of MCF7 cells grown in the absence or presence of elder flower extract (at effective EFE concentrations of 10 μg/mL, 50 μg/mL, and 100 μg/mL), after immuno-detection of ER-α (**A**) and PR (**B**); and presentation of the immunocytochemistry results by the semi-quantitative immunoreactivity score (IRS). Significantly different observations are highlighted with an asterisk.

MCF7 cells that were exposed to EFE flavonoids with the concentrations of 10 μg/mL, 50 μg/mL, and 100 μg/mL responded significantly with a downregulation of ER α at the concentrations of 10 μg/mL (3.5 ± 0.55) and 50 μg/mL (6.3 ± 0.88) compared to the control (11.33 ± 0.73, *p* = 0.002 and 0.004), (Figure 4A).

MCF7 cells that were exposed to EFE lignan and flavonoid extracts with the concentrations of 10 μg/mL, 50 μg/mL, and 100 μg/mL responded significantly in an upregulation of the PR in a dose-response pattern (Figure 4B). The upregulation of the progesterone IRS score significantly reached a peak at the EFE lignan concentration of 10 μg/mL (8 ± 0.98) compared to the control (3.3 ± 0.36, *p* = 0.002). As the EFE concentration increased, the IRS score decreased at 50 μg/mL to 7.66 ± 1.04, and at 100 μg/mL to 5.83 (Figure 4B). 

The same phenomenon was observed using EFE flavonoids where the IRS score increased from 2.66 ± 0.46 in the control to 6 ± 0 at 10 μg/mL (*p* = 0.002), and then decreased to 4.83 ± 0.59 (*p* = 0.026) at 50 μg/mL, and to 2.83 ± 0.44 at 100 μg/mL (Figure 3B).

### 3.5. Enterolactone Downregulates Expression of ERα and PR in a Dose-Response Pattern in MCF7 Cells

**Figure 5:**
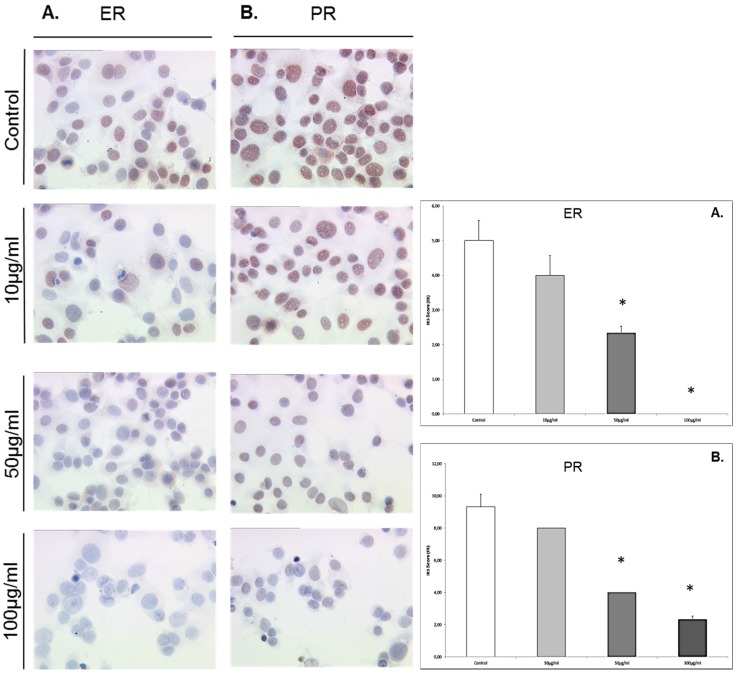
Representative microphotographs of MCF7 cells grown in the absence or presence of enterolactone at concentrations of 10 μg/mL, 50 μg/mL, and 100 μg/mL), after immuno-detection of ER-α (**A**) and PR (**B**); and presentation of the immunocytochemistry results by the semi-quantitative immunoreactivity score (IRS). Significantly different observations are highlighted with an asterisk.

MCF7 cells that were exposed to enterolactone at concentrations of 10 μg/mL, 50 μg/mL, and 100 μg/mL responded significantly with a downregulation of ER α at concentrations of 50 μg/mL (IRS score 2.5) and 100 μg/mL (IRS score 0) compared to the control (IRS score 5, *p* = 0.027 and 0.024) (see Figure 5). MCF7 cells that were exposed to enterolactone at concentrations of 10 μg/mL, 50 μg/mL, and 100 μg/mL responded with a dose-response-related downregulation of the PR. The downregulation of the PR was significant at enterolactone concentrations of 50 μg/mL (IRS score 4) and 100 μg/mL downregulation (IRS score 2) compared to the control (IRS score 9, *p* = 0.028 for both concentrations).

### 3.6. Enterodiol Downregulates Expression of ERα and PR Only at High Concentrations in MCF7 Cells

**Figure 6:**
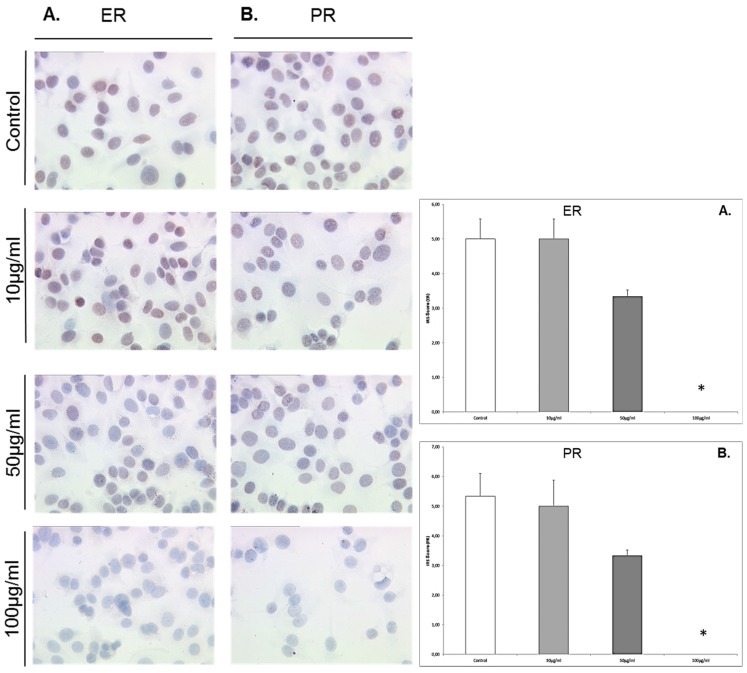
Representative microphotographs of MCF7 cells grown in the absence or presence of enterodiol at concentrations of 10 μg/mL, 50 μg/mL, and 100 μg/mL), after immuno-detection of ER-α (**A**) and PR (**B**) ; and presentation of the immunocytochemistry results by the semi-quantitative immunoreactivity score (IRS). Significantly observations are highlighted with an asterisk.

MCF7 cells that were exposed to enterodiol at concentrations of 10 μg/mL, 50 μg/mL, and 100 μg/mL responded with a significant downregulation of ERα only at 100 μg/mL (IRS score 0) compared to the control (IRS score 5, *p* = 0.023) (Figure 6). MCF7 cells that were exposed to enterodiol at concentrations of 10 μg/mL, 50 μg/mL, and 100 μg/mL responded with a significant downregulation of the PR at 100 μg/mL (IRS score 0) compared to the control (IRS score 5.5, *p* = 0.023).

### 3.7. Enterolactone Inhibits Estradiol Secretion in JEG-3 Cells and Induce Estradiol Secretion in BeWo and MCF7 Cells in a Dose-Response Pattern

**Figure 7:**
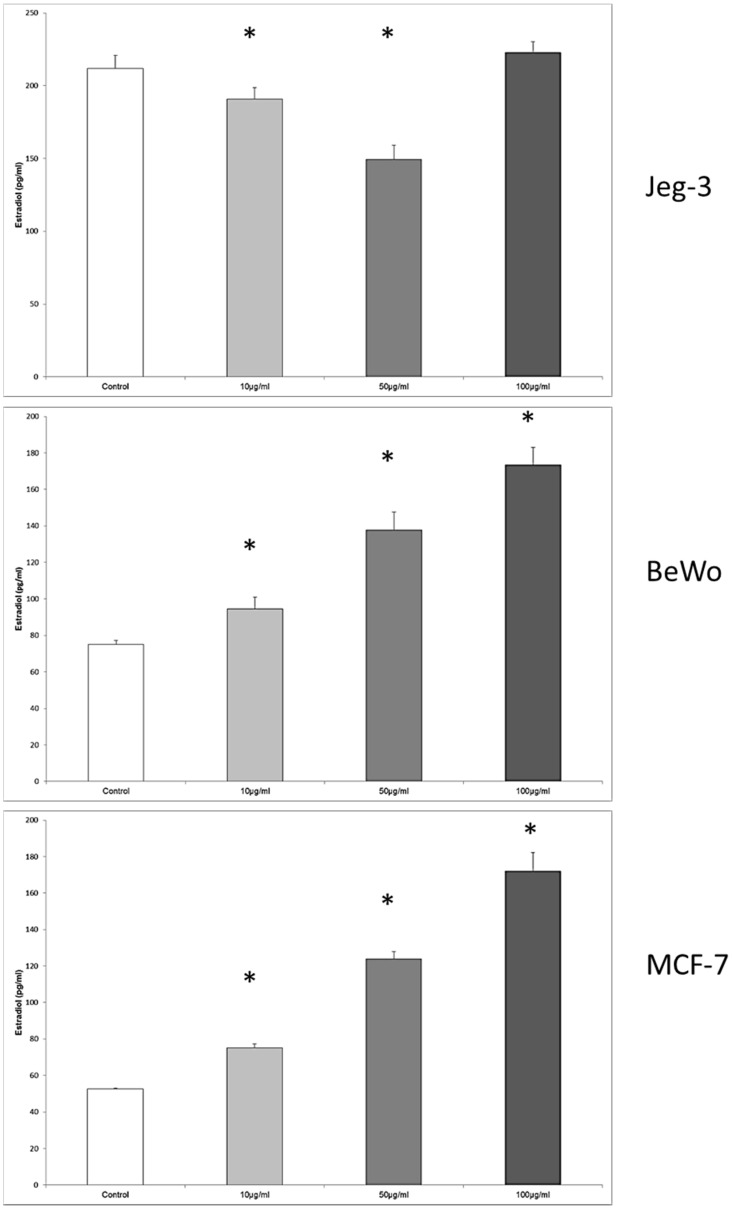
Estradiol concentration in the tissue culture medium of JEG-3, BeWo and MCF7 cells in the absence or presence of enterolactone. The effective enterolactone concentrations were 10 μg/mL, 50 μg/mL, and 100 μg/mL. Significantly different observation are highlighted with an asterisk.

In JEG-3 cells, the estradiol production was inhibited from 211.8 ± 8.88 pg/mL in the control to 190.9 ± 7.9 pg/mL, and 149.59 ± 7 pg/mL at enterolactone concentrations of 10 μg/mL and 50 μg/mL, *p* = 0.028, respectively (Figure 7).

The cultivation of the BeWo cell line with enterolactone resulted again in an upregulation of estradiol secretion from 75.07 ± 2.33 pg/mL in the control to 94.66 ± 6.39 pg/mL, 137.66 ± 10.04 pg/mL, and 173.53 ± 9.56 pg/mL when the enterolactone concentration was 10 μg/mL, 50 μg/mL, and 100 μg/mL. The differences between the stimulated cells and the control were significant at all concentration levels of enterolactone, *p* = 0.028, respectively. 

In MCF7 cells the concentrations of 10 μg/mL, 50 μg/mL and 100 μg/mL provoked an increased secretion of estradiol from 52.65 ± 7.90 pg/mL in the control to 75.22 ± 2.11 pg/mL at 10 μg/mL, 123.93 ± 3.93 pg/mL at 50 μg/mL, and 172.12 ± 10.05 pg/mL at 100 μg/mL, *p* = 0.028, respectively. 

### 3.8. Enterodiol Induces Estradiol Secretion in JEG-3, BeWo, and MCF7 Cells at Distinct Concentrations

**Figure 8:**
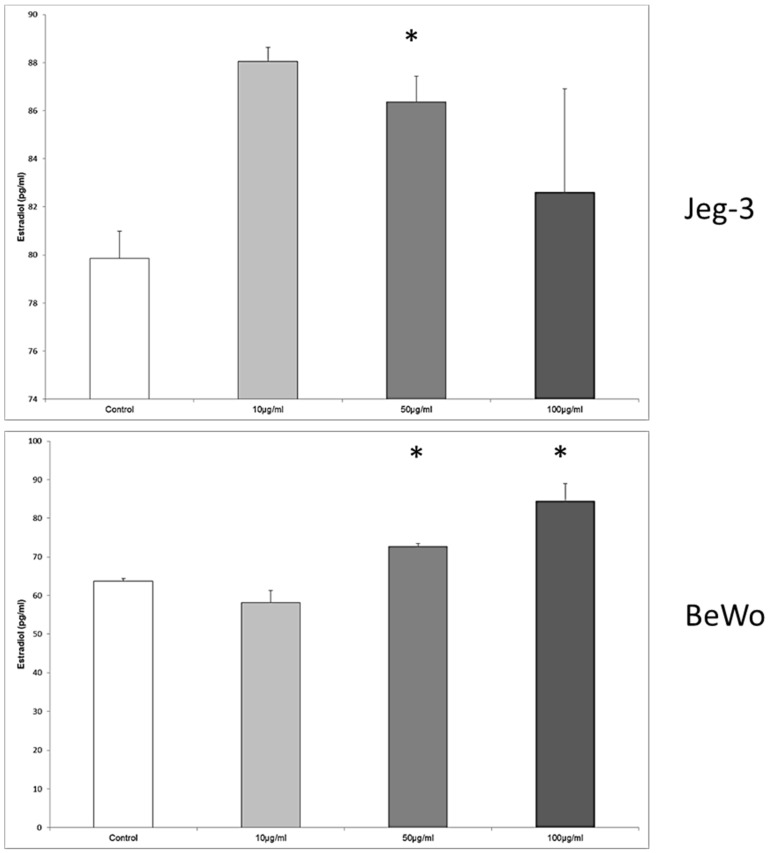

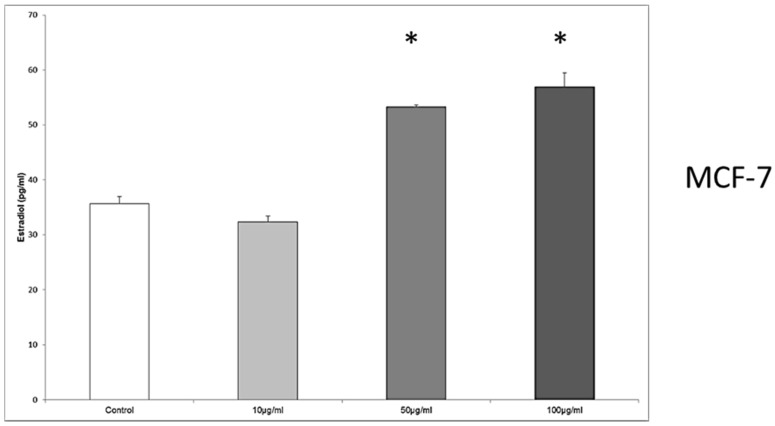
Estradiol concentration in the tissue culture medium of JEG-3, BeWo and MCF7 cells in the absence or presence of enterodiol. The effective enterodiol concentrations were 50 μg/mL and 100 μg/mL. Significantly different observation are highlighted with an asterisk.

In JEG-3 cells, the estradiol secretion was significantly enhanced from 79.85 ± 1.14 pg/mL in the control to 86.37 ± 1.07 pg/mL, when the concentration was 50 μg/mL enterodiol, *p* = 0.028 (Figure 8).

The cultivation of the BeWo cell line with enterodiol resulted again in a significant upregulation of estradiol secretion from 63.71 ± 0.68 pg/mL in the control to 72.71 ± 0.79 pg/mL, and 84.37 ± 4.63 pg/mL at the enterodiol concentrations of 50 μg/mL and 100 μg/mL, respectively. The differences between the stimulated cells and the control were significant at both concentration of enterodiol, *p* = 0.028, respectively. 

In MCF7 cells the concentrations of 50 μg/mL and 100 μg/mL provoked an increased secretion of estradiol from 35.64 ± 1.32 pg/mL in the control to 53.28 ± 0.39 pg/mL at 50 μg/mL, and 56.94 ± 2.54 pg/mL at 100 μg/mL, *p* = 0.028, respectively. 

## 4. Discussion

To our knowledge, this is the first study evaluating the phytoestrogen properties of EFE on BeWo, JEG-3, and MCF7 cells regarding the estrogen and progesterone response. Prior to this study it was uncertain if EFE contains phytoestrogen compounds. Although mass spectrometry proved that EFE contains lignans and isoflavones, the subgroups of each class were not identified and, thus, precision is lacking. EFE proved to be richer in lignans than in isoflavones (presented in Figure 1). This may explain why more significant results were found using the lignan EFE. However, further studies with isolated fractions of the subgroups of EFE lignans and isoflavones could clarify if one subgroup is more potent than the other. Therefore, it would be interesting to isolate and identify the different lignans and isoflavones in the EFE that cause phytoestrogen activity for further characterization. Before further evaluation in an animal model, in vitro evaluation of the various components’ effects as single substances is required.

In a previous study of our group, the phytoestrogen properties of pumpkin seed extract were tested on the same cells, which resulted in an unexpected estrogen secretion in all cell lines [18]. As hormone-dependent tumors react with proliferation when exposed to estrogens, pumpkin seeds, thus, could provoke carcinogenic effects. 

In contrast, EFE was the first of the potential phytoestrogens previously tested by our group, which had an inhibitory effect on the estradiol secretion of all three cell lines. 

The effect on JEG-3 and BeWo cells was observed to be dose-dependent. Interestingly, in MCF7 cells, estrogen secretion was higher following the administration of intermediate phytoestrogen concentrations than in controls or with the highest EFE concentration tested. The degree to which the inhibition of estrogen secretion results in a decreased cell proliferation has to be tested in further investigations using EFE. In addition, it is possible that at the highest EFE concentration estrogen secretion was decreased due to cytotoxic effects of the extract itself, as other studies suggest that phytoestrogens cause cytotoxicity and decrease growth in MCF7 tumors. For example, in a study by Bergman et al. [31] ovariectomized mice were treated with continuous release of estrogen. MCF7 tumors were established and mice were fed with basal diet or 10% flaxseed, and two groups that were fed basal diet received daily injections with enterodiol or enterolactone (15 mg/kg body weight). The regimens containing flax seeds or enterodiol or enterolactone injections resulted in decreased estrogen-induced growth and angiogenesis in solid tumors by decreasing the secretion of VEGF.

It is of interest that EFE induces not only an inhibition of estradiol secretion, but also an upregulation of the ERα in JEG-3 cells. It could be assumed that, if EFE causes an inhibition on the trophoblast estrogen secretion, the cells react by increasing ERα expression in order to obtain stimulation even in a low-estrogen environment. A recent study by Lim et al. [32] outlined that the flavonoid apigenin reduces survival of JEG-3 cells by inducing apoptosis via the PI3K/AKT and ERK1/2 MAPK pathways. Therefore, it seems likely that the phytoestrogens also found in EFE could trigger non-genomic estradiol receptor signal transduction causing apoptosis in JEG-3 cells. In contrast to the effects of EFE on JEG-3 cells, another study by our group [33] demonstrated that the two well-known phytoestrogens genistein and daidzein provoked a reduced progesterone production and a stimulation of the estrogen production in JEG-3 cells. Therefore, regarding the other extracts investigated by our group, the characteristics of EFE seem to be favorable for further research due to the properties of decreased estrogen secretion and increased ERα expression in JEG-3 cells.

MCF7 cells that were exposed to EFE extracts responded with a significant downregulation of ERα and an upregulation of PR, both predominantly in lower concentrations of EFE. Why the lower concentrations provoked a stronger effect on receptor expression remains unknown. Although it is, again, possible that higher concentrations of EFE resulted in cytotoxic effects leading to cell damage and, therefore, to decreased cellular function. Nevertheless, the fact that lower EFE concentrations resulted in a decreased expression of the ERα receptor and an increase in the progesterone receptor could be beneficial for clinical use as low blood concentrations of phytoestrogens are easier to achieve by dietary intake alone. It is important to mention that the concentrations used in this study were extremely high (non-physiological). The highest level of enterolactone that has been measured in serum/plasma in humans is 2 μmol/L (over 16 times less than the enterolactone concentration used). Furthermore, estradiol levels in adult females reach levels only as high as 300 pg/mL in the luteal phase (30,000 times less than the external control). Therefore, before realistic interpretation, our findings must be reevaluated in further studies using more physiologically relevant doses.

Our current findings partially concur with a previously-described downregulation of ER*α* and upregulation of PR on the MCF7 cells when treated with other potential phytoestrogen compounds such as flax and pumpkin isoflavone and lignan extracts or mixtures [19,34]. Interestingly, it has been demonstrated that estradiol has similar effects on the MCF7 ERα and on PR, as it causes a downregulation of ERα and an upregulation of PR [35,36]. Therefore, whether EFE causes MCF7 cell proliferation or inhibition has to be tested in future investigations. In a study by Stendahl et al., it was demonstrated that high progesterone receptor expression correlates with a better effect of adjuvant tamoxifen in premenopausal breast cancer patients [37]. This suggests clinical trial evaluation of elderflower as a combination partner for tamoxifen. 

It is unclear whether the lignans present in the EFE require any metabolic processing prior to exerting biological effects and whether the cell culture systems used are capable of completing this conversion. For example secoisolariciresinol diglycoside (SDG) is the primary lignan in flaxseed; however, in vitro studies use bioavailabile enterodiol and enterolactone when investigating effects of flaxseed lignans. This is because in vitro systems do not have the components necessary to convert SDG to enterodiol and enterolactone. Therefore, additional in vivo studies could provide valuable information regarding EFE metabolism prior to the conduction of further in vitro studies. Nevertheless, the pattern of hormone secretion and receptor expression of enerolactone and enterodiol tested on JEG-3, BeWo, and MCF7 cells were different to those of EFE. Therefore, it is probable that the lignans in EFE are not related to the enterolignans. Enterolactone and enterodiol in contrast to EFE inhibited not only the expression of the ER but also PR in MCF7 cells. Moreover contrary to EFE, both control substances upregulated estradiol production in BeWo and MCF7 cells in a concentration-dependent manner.

## 5. Conclusions

Our results clearly demonstrate beneficial features of EFE in the setting of hormone receptor-positive breast cancer MCF7 cells by inhibition of estrogen secretion, downregulation of Erα, and upregulation of PR. Decreased local and circulating estrogen concentrations are certainly considered an advantage in treating breast cancer. In that view, EFE could be related to reduced tumor cell proliferation, possibly suggesting a protective effect on breast cancer. Nevertheless, the results and the conclusions made must be interpreted with caution as this is an in vitro cell culture study. In this setting, the use of plant extracts instead of chemically pure agents may be advantageous as it may more accurately reflect the effects of phytoestrogen-rich diets.

If the effects of EFE can be attributed solely to potential phytoestrogen activity remains unsolved. To which degree other non-estrogenic pathways play a role can currently not be clarified. For example, mass spectrometry demonstrated a high amount of lipids in EFE. Lipids can inhibit cell proliferation through activation of PPARα and PPARγ (peroxisome proliferator-activated receptors) which bind as transcription factors to the retinoid X receptors and, thus, regulate the expression of various genes [38,39]. In MCF7 breast cancer cells PPARγ activates p53 by stimulating the transcription factor NFĸB (nuclear factor kappa-light-chain-enhancer of activated B-cells), which is a gene promoter of p53 and, thus, induces apoptosis [40]. Therefore, the following additional investigations are necessary to obtain further insight of the promising anti-carcinogenic effects of EFE: the results of hormone secretion and receptor expression of EFE should be correlated with DNA synthesis performance (BRDU proliferation assay), metabolic activity (MTT assay), and cytotoxicity (LDH assay) tests. Cytotoxicity could be evaluated in detail by immunohistochemistry or reverse transcriptase quantitative (RTQ)-PCR quantification of apoptosis-induced markers (for example, p53, p21, BCL2, Caspase 8/9). Then, as a possibility to determine the role of hormone receptor-mediated cell response, EFE could be tested on malignant ER-negative cells (e.g., BT-20). Furthermore, fractional chromatography could provide information of the individual substances and their impact on breast cancer cells. Finally, after further in vitro investigations, properly designed animal studies could highlight a potential role of EFE in trophoblast and breast cancer prevention and/or treatment. 

## Figures and Tables

**Table 1:** Antibodies used for expression analysis of steroid hormone receptors.

Salient Features of the Antibodies Used in the Present Study				
Antibody	(Source)	Origin	Dilution in PBS	Temperature
Anti-ERcr	(Dako, Germany)	Mouse monoclonal	1:150	1 h RT
Anti-ERβ	(Serotec, Germany)	Mouse monoclonal	1:600	O/N 4 °C
Anti-PR	(Dako, Germany)	Mouse monoclonal	1:50	1 h RT

ER = estrogen receptor; PR = progesterone receptor; O/N = overnight; RT = room temperature.
